# Diabetic nephropathy and hypertension in diabetes patients of sub-Saharan countries: a systematic review and meta-analysis

**DOI:** 10.1186/s13104-018-3670-5

**Published:** 2018-08-06

**Authors:** Fasil Wagnew, Setegn Eshetie, Getiye Dejenu Kibret, Abriham Zegeye, Getenet Dessie, Henok Mulugeta, Amanuel Alemu

**Affiliations:** 1 College of Health Science, Debre Markos University, Debre Markos, Ethiopia; 2 College of Health Science, University of Gondar, Gondar, Ethiopia; 3 Faculty of Medicine, The University of Queensland, Brisbane, Australia

**Keywords:** Diabetic nephropathy, Hypertension, Sub-Saharan countries

## Abstract

**Objective:**

This meta-analysis was undertaken to estimate the prevalence of diabetic nephropathy and its association with hypertension in diabetics of sub-Saharan African countries.

**Results:**

A total of 27 studies were included for the meta-analysis. The pooled overall prevalence of diabetic nephropathy was 35.3 (95% CI 27.46–43.14). In sub-group analyses by types of diabetes and regions, for instance, the prevalence was 41.4% (95% CI 32.2–50.58%) in type-2 diabetes mellitus and 29.7% (95% CI 14.3–45.1%) in Eastern Africa. Pooled point estimates from included studies revealed an increased risk of diabetic nephropathy with hypertension compared to without hypertension (OR = 1.67, 95% CI 1.31, 2.14). Diabetic nephropathy is a common complication in diabetic patients. Diabetic nephropathy complication is significantly higher in hypertensive patients. A preventive strategy should be adopted or planned to reduce diabetes mellitus and its complication of neuropathy, particularly in hypertensive.

**Electronic supplementary material:**

The online version of this article (10.1186/s13104-018-3670-5) contains supplementary material, which is available to authorized users.

## Introduction

Worldwide, around 387 million people have *diabetes mellitus* (DM) according to the International Diabetes Federation (IDF) update of 2014. Nearly 592 million people, or 1 person in 10, are anticipated to have diabetes in 2035 [1]. Diabetes is the single major cause of end-stage renal disease (ESRD) and consequent dialysis leads to a huge burden in terms of poor quality of life and economical costs [2].

*Diabetic nephropathy* (DN) is an emerging clinical and public health challenge and is related with adverse outcomes including ESRD and heart failure, as well as kidney replacement therapy [3, 4], deaths related to these terminal illnesses [5, 6] by compromising life expectancy [7] in most African countries in particular [3, 4]. Indeed, DN remains higher in African, Asians, and Native Americans as compared to Caucasians [8, 9]. Generally for the past two decades, the prevalence of DN in people with diabetes has not improved because of an increase in the prevalence of reduced eGFR [10]. Likewise, the magnitude of ESRD in DM patients decline faintly [11] and the rate of DN increase is significant perhaps due to higher rates of type 1 and type 2 DM [12, 13]. In Africa, the highest magnitude of DN is associated with late diagnosis, scarcity of screening and diagnostic resources, poor control of blood sugar and other precipitating factors, and inappropriate treatment [14–16].

Various factors may be associated with a worsening renal disease among diabetic patients. Some of the major factors include genetic predisposition [17, 18], improper control of blood sugar [19, 20] and hypertension [21, 22]. There is evidence that both diabetes mellitus and hypertension are extremely interconnected, and in majority of the cases cardiac consequence may be associated with advanced stages of DN. For example, the relationship between hypertension and DN can be explained by the retention of concentrated sodium and subsidiary blood vessel resistance [23]. Saying this, previous studies of the magnitude of DN are remains inconsistent and unclear. Furthermore, to our knowledge, there is no synthesis of existing contemporary evidence on the association between DN and hypertension in diabetic patients. Thus, the aim of this meta-analysis is to estimate the prevalence of DN and examine its association with hypertension among diabetic patients in sub-Saharan African countries.

## Main text

### Methods

#### Search strategy and study design

Using computerized databases, searches were performed to locate all studies on the prevalence of DN among diabetic patients in sub-Saharan countries. Databases included from EMBASE, Cumulative Index to Nursing and Allied Health Literature (CINHAL), Pub Med, MEDLINE, Google scholar and Google for grey literature. We extended our search by retrieving reference lists of eligible articles, hand searches for grey literature and other relevant literature collections. Observational studies conducted on the prevalence of DN among diabetic patients in sub-Saharan countries were selected for this meta-analysis. Search protocol was formulated by using such common key words as ‘Renal disease OR Renal insufficiency OR Diabetic nephropathy’ OR ‘DN’ OR end stage kidney failure, OR microalbuminuria AND hypertension OR HTN AND diabetes OR diabetes mellitus OR type 1 diabetes OR type 1 DM OR type 2 DM AND ‘sub-Saharan African countries (48 countries). We exhaustively searched by using the above key words in each sub-Saharan country, for instance, in Ethiopia, Botswana, Kenya and E.T.C. To make a certain scientific rigor, we strictly followed the preferred reporting of systematic reviews and meta-analysis (PRISMA) guideline [24].

#### Inclusion criteria

The study inclusion criteria included the following: those studies published in English, where the process of identifying DN was well described, and those studies with sufficient information to estimate the point prevalence of DN. The process of study inclusion is shown in Additional file 1: Fig. S1.

#### Exclusion criteria

Available studies were excluded if only the DN incidence in follow-up years was reported or when they did not explain the process/criteria of DN diagnosis and inability to access full-text.

#### Data extraction

All identified studies were screened for inclusion by two reviewers (FW and GDK). Discussions and mutual consensus were in place when possible arguments were raised between the two reviewers. These reviewers then assessed the full text of potentially eligible papers. We made some efforts to communicate primary authors whenever further information was needed. Numerator and denominator data and beta coefficients and their standard errors (if given) were used to calculate ORs, where ORs with 95% CI were not given. The data extraction format included first author, study year, region of study, study design, sample size, diagnostic criteria and types of diabetes. The occurrence of DN and HTN were also extracted from each included study.

#### Quality appraisal

The included articles were evaluated for quality, with only high quality studies included in the analysis. Two authors (FW, GDK) independently assessed the quality of each included paper. The reviewers compared their quality appraisal scores and resolved any disparity before calculating the final appraisal score. Newcastle–Ottawa Scale adapted for cross-sectional studies quality assessment tool was used [25]. The tool has three sections in general; the first section graded from five stars and due emphasis on the methodological quality of each original study. The second section of the tool explains with the comparability of the study. The third section focus on the outcome and statistical analysis of each original study. Articles with a scale of ≥ 6 out of 10 scales were considered as high quality. Consequently, all eligible studies had high quality scores.

#### Data analysis

Information about the study design, study sample, etc. were summarized by Microsoft excel. Relevant data were then exported to STATA/se version 14 software for analysis. Meta-analysis of pooled prevalence of DN was carried out using a random-effects model, generating a pooled prevalence with 95% CIs, by using the approach of DerSimonian and Laird statistical method [26]. Heterogeneity among studies was estimated using the Cochran’s Q and I^2^ statistic and is characterized as low, moderate, or high for 25%, 50%, and 75%, respectively [27]. We also scrutinized forest plots of summary estimates of each study to determine whether we could identify any outlier or heterogeneity. Publication bias was determined based on the symmetry of funnel plots [28], and Egger’s test [29]. Sub-group analyses were carried out by region, sample size and types of DM for there was significant heterogeneity across the included studies.

### Results

#### Flow chart

**Fig. 1:**
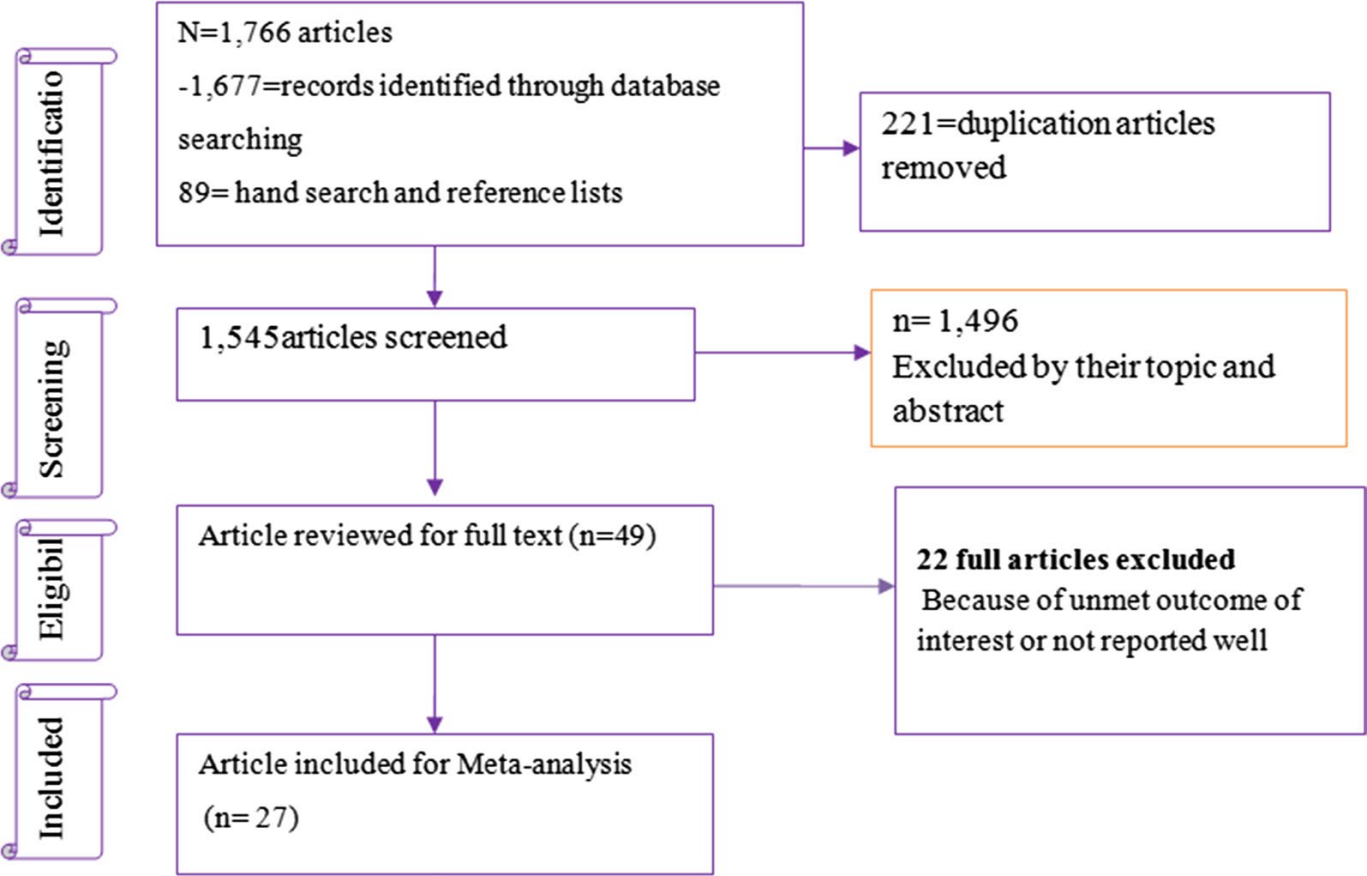
Pooled prevalence of DN among DM patients in sub-Saharan countries

Figure 1 shows the flow chart and selection process of exploring the prevalence of DN among DM patients. Our electronic database search offered 1766 articles, of those 1545 non-duplicate papers were assessed and 1496 excluded after reviewing their title and abstracts.

The remaining 49 were examined by full text. Of which, 22 articles were excluded due to unmet outcome of interest; only 27 studies met our inclusion criteria. The characteristics and quality assessment of all included studies are shown in Table 1. Five studies reported odds ratios of the odds of hypertension in diabetic patients. *Finally*, 27 articles representing 6552 participants met the inclusion criteria (Additional file 1: Fig. S1).

**Table 1:** Characteristics of included studies to determine the prevalence of DN and association with hypertension among diabetic patients in sub-Saharan countries

Authors name	Region	Country	Diagnostic criteria	Types of DM	Study design	Age of subjects	Sample size	No of people with outcome	Prevalence (%)
Rahlenbech [43]	Eastern Africa	Ethiopia	Microalbuminuria	Both types	Cross-sectional	≥ 18	170	55	32.35
Sobngwi et al. [44]	Central Africa	Cameroon	Microalbuminuria	Both types	Cross-sectional	≥ 18	64	34	53.13
Motala et al. [45]	Southern Africa	South Africa	Proteinuria	Both types	Cross-sectional	All	219	54	24.66
Wanjohi et al. [46]	Eastern Africa	Kenya	Albuminuria	Type 2	Cross-sectional	≥ 18	100	26	26.00
Rotchford and Rotchford [31]	Southern Africa	South Africa	Microalbuminuria	Both types	Cross-sectional	≥ 18	254	102	40.16
Albiosu [47]	Western Africa	Nigeria	Microalbuminuria	Both types	Cross-sectional	≥ 18	342	97	28.36
Alebiosu et al. [48]	Western Africa	Nigeria	Any sign	Both types	Cross-sectional	Not report	465	191	41.08
Agaba et al. [49]	Western Africa	Nigeria	Microalbuminuria	Type-2	Cross-sectional	≥ 18	65	32	49.23
Mafundikwa et al. [50]	Eastern Africa	Zimbabwe	Proteinuria	Type-2	Cross-sectional	≥ 18	75	16	21.33
Lutale et al. [51]	Eastern Africa	Tanzania	Microalbuminuria	Both types	Cross-sectional	All age	244	26	10.66
Majaliiwa et al. [52]	Eastern Africa	Tanzania	Microalbuminuria	Type-1	Cross-sectional	< 18	99	29	29.29
Rahamtalla et al. [53]	Eastern Africa	Sudan	Nephropathy	Type-2	Cross-sectional	≥ 18	58	26	44.83
Rasmussen et al. [54]	Eastern Africa	Zambia	Microalbuminuria	Not-report	Cross-sectional	< 18	193	24	12.44
Tamba et al. [55]	Central Africa	Cameroon	Nephropathy	Type-2	Cross-sectional	≥ 18	140	35	25.00
Janmohamed et al. [56]	Eastern Africa	Tanzania	eGFR	Both types	Cross-sectional	≥ 18	369	308	83.47
Ajayi et al. [57]	Western Africa	Nigeria	eGFR	Type 2	Cross-sectional	≥ 18	628	242	38.54
Deribe et al. [58]	Eastern Africa	Ethiopia	eGFR	Not report	Cross-sectional	≥ 18	216	19	8.80
Fiseha et al. [59]	Eastern Africa	Ethiopia	eGFR	Both types	Cross-sectional	≥ 18	214	51	23.83
Bunza et al. [60]	Western Africa	Nigeria	Nephropathy	Both types	Cross-sectional	≥ 18	100	22	22.00
Ngassa et al. [61]	Southern Africa	South Africa	Microalbuminuria	Both types	Cross-sectional	≥ 18	754	251	33.29
Diouf et al. [62]	Western Africa	Senegal	Microalbuminuria	Type-2	Cross-sectional	≥ 18	195	95	48.72
Chukwuani et al. [63]	Western Africa	Nigeria	Microalbuminuria	Type-2	Cross-sectional	≥ 18	200	76	38.00
Bekele [64]	Eastern Africa	Ethiopia	Nephropathy	Both types	Cross-sectional	≥ 18	355	68	19.15
Ufuoma et al. [65]	Western Africa	Nigeria	Microalbuminuria	Type-2	Cross-sectional	≥ 18	200	116	58.00
Machinngura et al. [66]	Eastern Africa	Zimbabwe	Nephropathy	Both types	Cross-sectional	≥ 18	344	154	44.77
Radikara [67]	Southern Africa	Botswana	Nephropathy	Type-2	Cross-sectional	≥ 18	408	259	63.48
Marie et al. [30]	Central Africa	Cameroon	Microalbuminuria	Not report	Cross-sectional	≥ 18	81	28	34.57

#### Characteristics of included studies

A total of 6552 participants were represented by 27 included studies (Table 1), published from 1997 to 2017. Ten studies (37%) reported the prevalence of DN among type-2 diabetic patients. Three studies (11.1%) included all age groups (both children and adults); study exclusively assessed DN among type-1 DM (Table 1).

Five studies included determined the effect of hypertension on diabetic nephropathy. These studies reported ORs between 1.4 [30] and 2.11 [31] for the rate of hypertension leading to DN (Additional file 2: Table S1).

#### Meta-analysis

As presented in the forest plot (Fig. 1), the pooled prevalence of DN among diagnosed DM cases was 35.3 (95% CI 27.46–43.14). The I^2^ test result indicated high heterogeneity (I^2^ 98.2%, p < 0.001). Thus, a subgroup analysis was done.

**Fig. 2:**
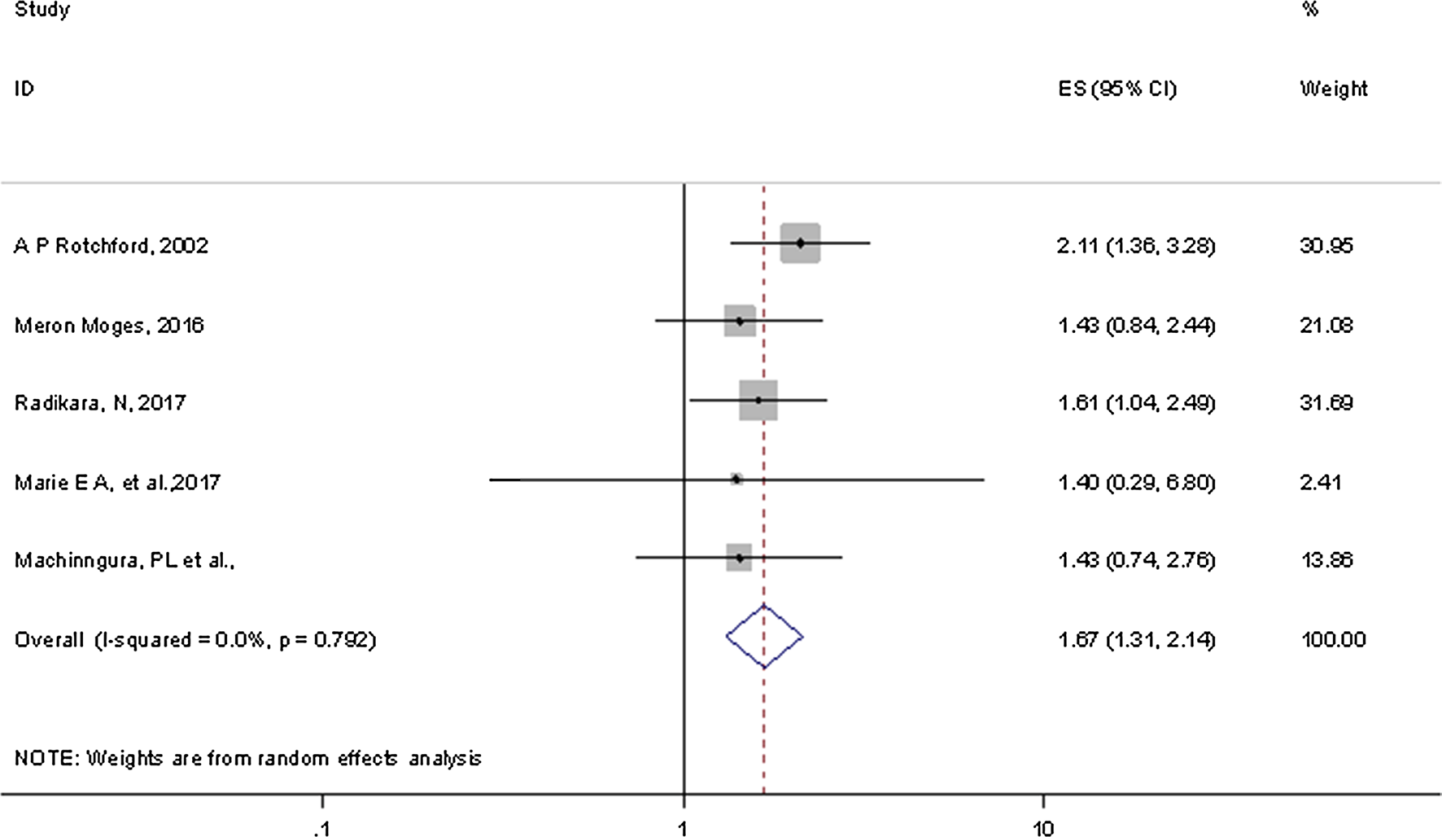
Pooled odds ratio indicating the association of hypertension with DN in diabetic patients

Our meta-analysis of the association between DN and hypertension included five studies, all of which reported ORs explicitly. Pooled point estimates from cross-sectional studies showed an increased risk of DN with hypertension compared without hypertension (OR = 1.67, 95% CI 1.31, 2.14). Overall heterogeneity of these studies was not significant (I^2^ = 0.0%, p = 0.79) (Fig. 2).

### Discussion

DN is becoming universal cause of ESRD and is recognized as an independent risk factor for cardiovascular disease [32]. Once elevated urinary albumin excretion, it may be inevitable to end with the development of nephropathy, though it may be possible to significantly hinder its development at early stages of the disease. The roles of glucose and blood pressure control may suffice here.

As shown in Additional file 3: Fig. S2, the burden of DN in diabetic patients has been significantly increased from 2015 to 2017. Therefore it needs special attention devotion to minimize the occurrence of the disease.

To our knowledge, this is the first pooled analysis of the prevalence of DN and its association with hypertension in sub-Saharan African countries. Existing evidence from included studies suggest that DN is high among diabetic patients in sub-Saharan countries. It is a study done by Stanford A [33] that revealed adults with type-2DM (n = 2006), with 38.3% having had DN during 2007–2012. Likewise, of the 5072 confirmed diabetes diagnosis, 31% had clinically significant DN, (D2) with microalbuminuria of 31.6 (95% CI 30.6–32.6) [34]. On the other hand, this finding is higher as compared to previous systematic review and meta-analysis done by Elhafeez et al. [35] reporting 24.7% (95% CI 23.6–25.7%) pooled prevalence of DN among diabetic patients [35]. This discrepancy attributed to differences in some factors including study period, sociodemographic characteristics, diagnostic criteria, as well as the methods of measurement of proteinuria and urine collection, diabetic type and duration and varying prevalence of hypertension across studies might contribute for the difference.

We did sub-group analysis due to a significant high heterogeneity. As showed in Additional file 4: Table S2, the prevalence of DN was higher in type-2 DM [41.39% (95% CI 32.2–50.6)] and Southern Africa [40.4% (95% CI 24.1–56.7)], correspondingly (Additional file 4: Table S2).

This study also revealed that hypertension significantly increased the occurrence of DN among people who have a diagnosis of DM. This finding supported by study done Wu B showed that hypertension is a major factors associated with CKD among diabetes patients (OR = 1.78) [33]. Likewise, another study revealed that hypertension is considered to pivotal role for the occurrence of DN [36]. Furthermore, the association between increased blood pressure and DN was recognized by most of the previous studies [37–39]. This could be due to oxidative stress and inflammation process. That is, the evidence suggests local generation of oxidative stress and inflammation is common mechanism in DN pathogenesis in the presence of hypertension and DM. Furthermore, hypertension induced increased intra-glomerular pressure leads to glomerular sclerosis and different renal diseases [40–42].

The overall Egger’s test for publication bias revealed no statistically significant evidence, *p* value = 0.08.

### Conclusion

DN is an ordinary complication in diabetic patients where pooled point estimates showing an increased risk of DN with hypertension. We recommend that a multifactorial approach, including lifestyle modification, Early detection of microalbuminuria, blood glucose control, blood pressure normalization, and the use of treatment that hold up with the RAS (Rennin Angiotensin System) and oxidative stress.

## Limitations

However, the findings need to be considered in the context of some important limitations. These include the inclusion of studies published only in English that may cause language bias. Furthermore, due to significant heterogeneity across studies, this study did not determine other possible risk factors contributing for the occurrence of DN in DM patients.

## Additional files

**Additional file 1: Fig. S1.** Flow chart describing selection of studies for a meta-analysis of the prevalence of DN and association hypertension among diabetic patients in sub-Saharan Africa.

**Additional file 2: Table S1.** The effect of hypertension on diabetic nephropathy among diabetes patients.

**Additional file 3: Fig. S2.** Time trend of DN in sub-Saharan countries from 1997 to 2017.

**Additional file 4: Table S2.** Subgroup analysis based on region, types of diabetes and sample size among diabetes patients.

## Abbreviations

CKD: chronic kidney disease
DN: diabetic nephropathy
DM: diabetes mellitus
eGFR: estimated glomerular filtration rate
ESRD: end-stage renal disease
HTN: hypertension
OR: odds ratio
