# Supplementary figures and images for: Diabetic nephropathy and hypertension in diabetes patients of sub-Saharan countries: a systematic review and meta-analysis
Source: BMC Res Notes. 2018 Aug 6;11:565. doi: 10.1186/s13104-018-3670-5 (PMC6080368; doi:10.1186/s13104-018-3670-5)

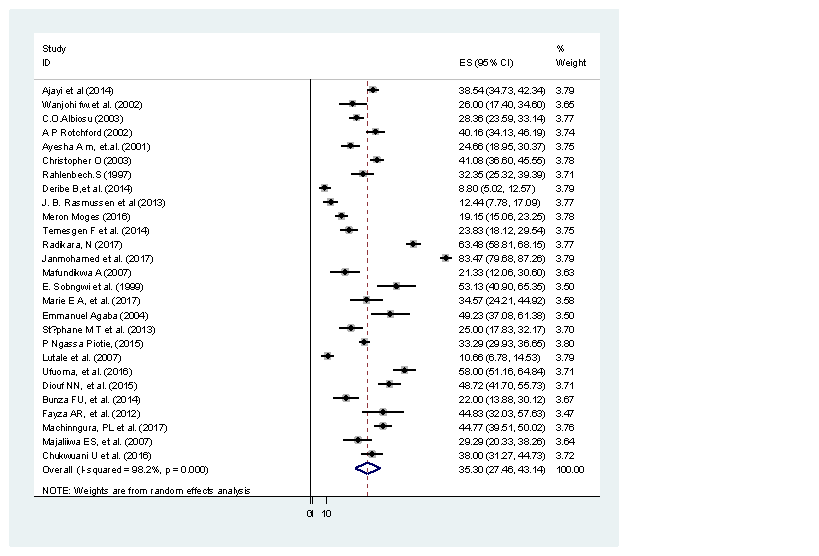

Supplement: Supplementary file 1 — Additional file 1: Fig. S1. Flow chart describing selection of studies for a meta-analysis of the prevalence of DN and association hypertension among diabetic patients in sub-Saharan Africa. [file 13104_2018_3670_MOESM1_ESM.png]

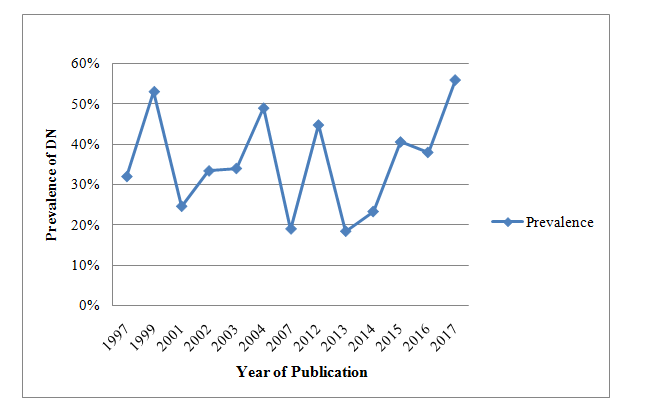

Supplement: Supplementary file 3 — Additional file 3: Fig. S2. Time trend of DN in sub-Saharan countries from 1997 to 2017. [file 13104_2018_3670_MOESM3_ESM.png]
