# Supplementary material for: Diabetic nephropathy and hypertension in diabetes patients of sub-Saharan countries: a systematic review and meta-analysis
Source: BMC Res Notes. 2018 Aug 6;11:565. doi: 10.1186/s13104-018-3670-5 (PMC6080368; doi:10.1186/s13104-018-3670-5)
Supplement: Supplementary file 2 — Additional file 2: Table S1. The effect of hypertension on diabetic nephropathy among diabetes patients. [file 13104_2018_3670_MOESM2_ESM.docx]

Table S1: The effect of hypertension on diabetic nephropathy among diabetes patients

| **Authors** | **Regions** | **Variables** | **DN** | **Non-DN** | **OR(Odds)** |
| --- | --- | --- | --- | --- | --- |
| Rotchford, 2002 [[34](file:///D:\new%20BOOK%20AND%20RES\manuscripts%20@@\DN\Revised%20version%20of%20Manuscript.docx#_ENREF_34)] | South Africa | HTN  Non-HTN | 165  88 | 44  96 | 2.11  1 |
| Bekele, 2016 [[52](file:///D:\new%20BOOK%20AND%20RES\manuscripts%20@@\DN\Revised%20version%20of%20Manuscript.docx#_ENREF_52)] | Ethiopia | HTN  Non-HTN | 38  30 | 129  146 | 1.43  1 |
| Radikara, N, 2017[[55](file:///D:\new%20BOOK%20AND%20RES\manuscripts%20@@\DN\Revised%20version%20of%20Manuscript.docx#_ENREF_55)] | Botswana | HTN  Non-HTN | 189  70 | 98  51 | 1.61  1 |
| Marie E A, et al.,2017[[56](file:///D:\new%20BOOK%20AND%20RES\manuscripts%20@@\DN\Revised%20version%20of%20Manuscript.docx#_ENREF_56)] | Cameroon | HTN  Non-HTN | 3  23 | 4  43 | 1.4  1 |
| Machinngura, et al.,2017[[54](file:///D:\new%20BOOK%20AND%20RES\manuscripts%20@@\DN\Revised%20version%20of%20Manuscript.docx#_ENREF_54)] | Zimbabwe | HTN  Non-HTN | 163  27 | 138  16 | 1.43  1 |
|  |  |  |  |  |  |

HTN: Hypertension
