# Supplementary material for: Diabetic nephropathy and hypertension in diabetes patients of sub-Saharan countries: a systematic review and meta-analysis
Source: BMC Res Notes. 2018 Aug 6;11:565. doi: 10.1186/s13104-018-3670-5 (PMC6080368; doi:10.1186/s13104-018-3670-5)
Supplement: Supplementary file 4 — Additional file 4: Table S2. Subgroup analysis based on region, types of diabetes and sample size among diabetes patients. [file 13104_2018_3670_MOESM4_ESM.docx]

Table S2: Subgroup analysis based on region, types of diabetes and sample size among diabetes patients

| **Variables** | **Characteristics** | **Number of studies** | **Estimates with 95% CI** | **P-value** |
| --- | --- | --- | --- | --- |
| **Types of DM** | Type-1 DM | 1 | 29.3(20.3-38.25) | <0.001 |
|  | Type-2 DM | 10 | 41.39(32.19-50.58) |  |
|  | Both Type 1&2 | 13 | 35.09(23.1-47.1) |  |
| **Region** | Western Africa | 8 | 40.2(33.3-47.21) | <0.001 |
|  | Eastern Africa | 12 | 29.7(14.28-45.1) |  |
|  | Southern Africa | 4 | 40.4(24.11-56.7) |  |
|  | Central Africa | 3 | 35.3(27.46-43.14) |  |
| **Sample size** | <250 samples | 18 | 30.8(23.7-38) | <0.001 |
|  | >250 samples | 9 | 43.5(29.9-57.2) |  |
